# Experiments and Model for Serration Statistics in Low-Entropy, Medium-Entropy, and High-Entropy Alloys

**DOI:** 10.1038/srep16997

**Published:** 2015-11-23

**Authors:** Robert Carroll, Chi Lee, Che-Wei Tsai, Jien-Wei Yeh, James Antonaglia, Braden A. W. Brinkman, Michael LeBlanc, Xie Xie, Shuying Chen, Peter K. Liaw, Karin A. Dahmen

**Affiliations:** 1 University of Illinois at Urbana-Champaign, Department of Physics, 1110 West Green Street, Urbana, IL 61801, USA; 2 National Tsing Hua University, Department of Materials Science and Engineering, Hsinchu, 30013, Taiwan; 3 The University of Tennessee-Knoxville, Department of Materials Science and Engineering, Knoxville, TN 37996, USA.

## Abstract

High-entropy alloys (HEAs) are new alloys that contain five or more elements in roughly-equal proportion. We present new experiments and theory on the deformation behavior of HEAs under slow stretching (straining), and observe differences, compared to conventional alloys with fewer elements. For a specific range of temperatures and strain-rates, HEAs deform in a jerky way, with sudden slips that make it difficult to precisely control the deformation. An analytic model explains these slips as avalanches of slipping weak spots and predicts the observed slip statistics, stress-strain curves, and their dependence on temperature, strain-rate, and material composition. The ratio of the weak spots’ healing rate to the strain-rate is the main tuning parameter, reminiscent of the Portevin-LeChatellier effect and time-temperature superposition in polymers. Our model predictions agree with the experimental results. The proposed widely-applicable deformation mechanism is useful for deformation control and alloy design.

A series of new tensile deformation experiments is presented, which are performed at slow strain-rates on metals ranging from conventional alloys (i.e., with fewer than five principal elements) to high-entropy alloys (HEAs)^1^,^2^,^3^,^4^,^5^,^6^,^7^,^8^,^9^,^10^,^11^,^12^,^13^,^14^,^15^,^16^,^17^,^18^. HEAs are new alloys that contain at least five principal elements of roughly-equal proportion. They are high-performance materials with excellent properties for elevated-temperature applications, such as boilers and turbines^1^,^2^,^3^,^4^,^5^,^6^,^7^,^8^,^9^,^10^,^11^,^12^,^13^,^14^,^15^,^16^,^17^,^18^. In a certain temperature and strain-rate regime, HEAs deform via sudden slips that are visible as stress drops or “serrations” in the stress-strain curves. These slips can make it difficult to precisely control the deformation behavior. Understanding how their sizes can be reduced could be used to smoothen the deformation properties, and, thus, make the deformation process more predictable and easier to control.

Here we present new experimental results on alloys exposed to slow tensile deformation. We compare the results to the predictions of an analytic model that explains the observed serration statistics and the time-series properties of the slips. The model predicts how the scaling behavior of the slip-size distribution and related statistical properties can be changed by tuning temperature, strain-rate, or alloy composition. The experimental results agree with the model predictions. As explained below, both the experiments and the model show that the deformation mechanism of HEAs differs from that of conventional alloys. In the following sections, we discuss (1) the experiments, (2) the model, its predictions and applicability range, and (3) the comparison of the experimental results to the model predictions. Potential applications of the newly-proposed deformation mechanisms, include materials testing, evaluation, and the design of new alloys with improved deformation properties.

## Experimental Procedure

### Materials used in the experiments

We perform experiments on HEAs^1^,^2^,^3^,^4^,^5^,^6^,^7^,^8^,^9^,^10^,^11^,^12^,^13^,^14^,^15^,^16^,^17^,^18^, which are new materials with important structural and functional applications. They possess great toughness^1^, high-temperature softening resistance^19^, large ductility^19^,^20^,^21^, good fatigue resistance^10^,^22^, and excellent wear resistance^23^,^24^. They consist of at least five principal elements in equimolar or near-equimolar ratios.

HEAs are defined as alloys that have a mixing entropy higher than 1.5R, where R is the gas constant. In addition, medium-entropy alloys (MEAs) are defined as having the mixing entropy between 1R and 1.5R, and low-entropy alloys (LEAs) as being below 1R. Most conventional alloys are LEAs. However, some stainless steels, superalloys, and bulk metallic glasses are MEAs because of their high concentrations of alloying elements.

In our experiments, a series of metals, containing Ni, CoNi, CoFeNi, CoCrFeNi, and CoCrFeMnNi, were designed specifically because they all have a solo face-centered-cubic (FCC) structure, thus providing a common basis for the comparison of serration phenomena. Their mixing entropies, approximately represented by the configurational entropy, are 0R, 0.69R, 1.1R, 1.39R, and 1.61R, respectively. With this range of entropies, the alloy series covers LEAs, MEAs, and HEAs. We observe changes in the serration phenomena from FCC-LEAs to FCC-HEAs, as temperature is tuned. As we will show, the measurements confirm the predicted effects on the serration statistics, of tuning temperature, strain-rate, and alloy composition.

### Design of Experiments

In our experiments, the five metals, Ni, CoNi, CoFeNi, CoCrFeNi, and CoCrFeMnNi, were slowly deformed under tension at different strain-rates from 1 × 10^−5^/s to 1 × 10^−2^/s and temperatures from 300 °C to 700 °C. Each experiment was repeated 3 to 4 times to ensure the reliability of the test results. The slip statistics were extracted from the measured stress-time curves and, then, compared with the new predictions of the above statistical model. Details on the experiments are given in the Methods Section.

## Results

**Figure 1:**
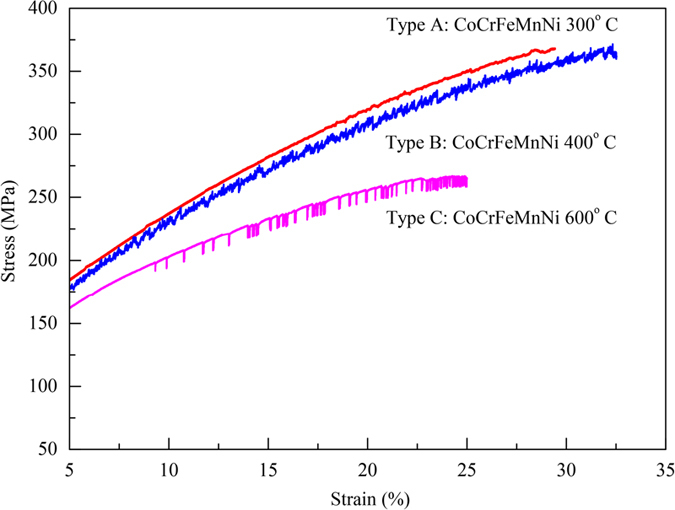
Close-ups of experimentally-measured stress-strain curves at a strain-rate of 10^−4^/s. (Stress drops are the serrations.) (**a**) Type-A example from CoCrFeMnNi, 300 °C. (**b**) Type-B example from CoCrFeNi, 400 °C. (**c**) Type-C example from CoCrFeNi, 600 °C. Type-A curves show abrupt rises, followed by stress drops (serrations) at various points of the stress-strain curve. Type-A bands are observed in the low-temperature (high strain-rate) part of the dynamic strain aging (DSA) regime. Type-B stress-strain curves oscillate in quick succession about an average trend [see supplementary information (SI)]. Type-B bands occur at higher temperatures and lower strain-rates of the DSA regime and also develop from type A with increasing strain. Type-C curves show large stress drops (serrations). They occur at higher temperatures and lower strain-rates than types A and B. (The SI shows the same data with dashed lines indicating the average trend of the stress-strain curves for comparison.)

Tensile test results have shown that the present FCC alloy series of CoCrFeMnNi have good elongation between 15% and 50% in the range of test temperatures and strain-rates. Table 1 lists the results of all samples tested at the strain-rate of 1 × 10^−4^/s, and indicates the occurrence or nonoccurrence of serration behavior. Among the 31-analyzed samples at different temperatures, 14 samples display sudden slips, or stress-drops, called “serrations”, which are reminiscent of the Portevin-LeChatellier (PLC) effect that is well studied for conventional materials^25^. As explained below, the time series properties of the serrations resemble those of conventional alloys with type-A, B, or C PLC-band behavior^25^. Here we briefly summarize features of the PLC-bands that are immediately relevant for our analysis. As shown in Fig. 1, type-C PLC-band behavior^25^,^26^,^27^,^28^,^29^,^30^,^31^,^32^,^33^,^34^,^35^,^36^,^37^,^38^,^39^,^40^,^41^ is characterized by almost periodically-recurring large serrations, type-B PLC-band behavior is usually characterized by smaller system-spanning (*large*) serrations that occur less regularly than in the type-C PLC-bands, along with *smaller* slips that may have a fairly-broad size distribution, and type-A PLC-bands^25^,^26^,^27^,^28^,^29^,^30^,^31^,^32^,^33^,^34^,^35^,^36^,^37^,^38^,^39^,^40^ are marked by irregularly-occurring smaller slips that are broadly distributed in size. The supplementary material provides a summary of additional information from the literature^25^,^26^,^27^,^28^,^29^,^30^,^31^,^32^,^33^,^34^,^35^,^36^,^37^ about these different deformation behaviors observed in conventional alloys.

Table 1 shows the observed PLC-band types that were identified by comparison with the classification of types-A to E in the literature^25^,^26^,^27^,^28^,^29^,^30^,^31^,^32^,^33^,^34^,^35^,^36^,^37^. Figure 1 shows the examples of stress-strain curves featured with types-A, B, and C PLC-bands.

In Table 1, at the strain-rate of 1 × 10^−4^/s, the Ni metal and CoNi alloy do not display serrations from 300 °C to 700 °C. The CoFeNi alloy has serrations from 400 °C to 500 °C. CoCrFeNi has serrations from 300 °C to 600 °C. CoCrFeMnNi has serrations from 300 °C to 620 °C. Note that over the temperature ranges we explored, we observed serrations only in the ternary, quaternary, and quinary alloys. Additionally, the temperature range of serrations broadens, as the number of elements increases. As for the evolution of serration types, the quinary alloy displays the whole span evolving from type A at low temperatures to type B at medium temperatures and to type C at high temperatures, which is consistent with the general observation from conventional alloys^25^. CoFeNi and CoCrFeNi do not show the whole span from types-A to C. The reason is likely the limited selective testing temperature.

**Figure 2:**
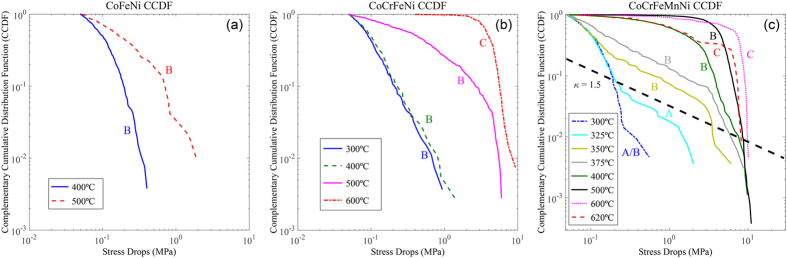
(**a**) Complementary cumulative avalanche-size-distribution function, C(S), for CoFeNi samples with PLC behavior. (**b**) Size-distribution function for CoCrFeNi samples with PLC behavior. (**c**) Size-distribution function of CoCrFeMnNi samples with PLC behavior. The PLC-band classifications for each temperature appear near the curve in the same color. The straight-dashed black line indicates a power-law with the predicted exponent, *κ* = 1.5, in close agreement with the data (see text).

To quantify the serration statistics, Figure 2 shows the associated complementary cumulative distributions of slip sizes, *C*(*S*), which gives the fraction of serrations that are larger than size, *S*. Here *S* is defined as the total stress drop observed during a serration. In the following sections, we first describe a simple model that explains (1) the observed time series properties of the serrations (*i.e*., type-A, B, or C PLC-bands) and (2) the observed scaling behavior of the serration-size distributions and their dependence on temperature, strain-rate, and composition.

### The Model

#### Model Assumptions

The simple coarse-grained model^42^ assumes that materials have weak spots. For example, in crystals, the weak spots are dislocations. Under slow deformation, a weak spot slips, when the local stress exceeds a random local failure stress. Through the elastic coupling, it can thereby trigger other weak spots to slip also, resulting in a slip avalanche, also known as serration. Weak spots re-stick when the local stress drops below a local arrest stress imposed by obstacles along the slip planes.

If the material has *N* lattice points, the local stress, τ_*l*_, at a lattice point, *l*, is given by^42^


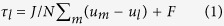


where *F* is the applied stress, *J* is the elastic-coupling constant, and *u*_*m*_ is the cumulative slip of the weak spot at the site, *m*. Each point fails, when the local stress, τ_*l*_, exceeds its local failure threshold (slip stress), *τ*_*f,l*_. When site, *l*, fails, it slips by an amount, Δ*u*_*l*_, thereby reducing the stress to a sticking stress, *τ*_*a,l*_. The associated stress drop is *τ*_*f,l*_ − *τ*_*a,l*_ ~ *2G* Δ*u*_*l*_, where *G* ~ *J* is the elastic shear modulus.

For HEAs, the model assumes that weak spots are weakened even further upon slipping to model how dislocations break free from the surrounding pinning atoms. This assumption is reminiscent of the Portevin-LeChatellier (PLC) effect or dynamic strain aging^25^,^30^,^38^,^39^,^40^,^41^. The weak spots re-heal to their original strength during the time between avalanches at a material-specific healing rate, *Λ*. Specifically, after a weak spot has slipped, the local failure stress, *τ*_*f,l*_, drops from its initial value, *τ*_*s,l*_, to a lower value, *τ*_*d,l*_, by an amount given by the weakening parameter, *ε* 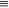 (*τ*_*s,l*_ − *τ*_*d,l*_)*/τ*_*s,l*_. It remains at this weakened value until the slip avalanche is completed. Afterwards, *τ*_*f,l*_ is increased to its initial value, *τ*_*s,l*_, at the temperature-dependent healing rate, *Λ*, until the weak spot slips again during a new avalanche, and the process repeats.

The re-strengthening of the weak spots models the rearrangement of atoms around arrested dislocations, leading to the (re-)“pinning” or anchoring of stationary dislocations in place. The weakening process models the dislocation breaking free from the pinning atoms around it. While the dislocation is free, it can slip more easily; when it meets obstacles, it is pinned again.

As we discuss below, for the alloys discussed here, the weakening, ε, depends on temperature and strain-rate. (Note that the weakening here is not primarily reflecting the overall strength of the weak spots, but a measure of how much a spot weakens through a slip, relative to its strength just before an avalanche.)

**Figure 3:**
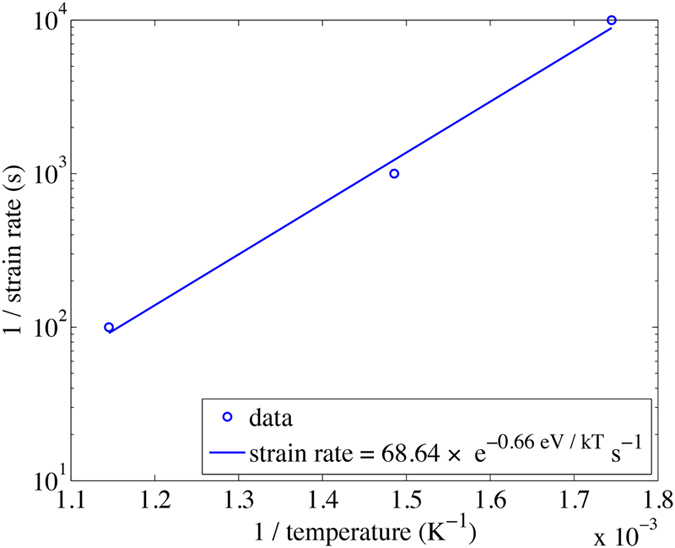
Testing for Arrhenius-activation processes and time-temperature superposition by comparing the data to the predictions of Equation (5) for CoCrFeMnNi. Here *k* = 8.617 × 10^−5^ eV/K is Boltzmann’s constant. As seen in Table 2, the temperature, T, and strain-rate, Ω, of the shown three data points lead to similar slip statistics, with type-A band behavior close to the transition to the type-B characteristics (see Table 2). The data points extracted from Table 2 are (Ω, T) = (10^−4^/s, 300 °C), (Ω, T) = (10^−3^/s, 400 °C), and (Ω, T) = (10^−2^/s, 600 °C). The data points fall on a straight line within the accuracy of the step sizes of the temperature measurements, consistent with the prediction of Equation (5) of the model.

We show below that our experiments on HEA can be modeled by a weakening, *ε*, that grows with the ratio, *Λ/Ω*, of the healing rate, *Λ*, relative to the strain-rate, Ω. For sufficiently-low temperatures, it is reasonable to assume that pinning of the dislocations involves activated processes. Consequently, the healing rate, *Λ*, follows an Arrhenius-like temperature (*T*) dependence, *Λ* ~ exp(−*B/T*), where *B* is a material-dependent constant. In close analogy to a similar mechanism in polymers, known as time-temperature superposition^43^, one concludes that the relevant parameter for the slip statistics is then *Λ/Ω* ~ exp(−*B/T*)/*Ω, i.e., similar slip statistics are expected for temperatures*, *T*, *and strain-rates*, *Ω*, *for which* exp(−*B/T*)/*Ω* = *constant.* As discussed in the experimental section, this prediction is consistent with the experimental data (Figures 1, 2, 3).

#### Comparison to conventional alloys

As mentioned above, a similar weakening effect is observed in conventional materials with dynamic-strain aging (DSA) for the PLC effect^25^,^30^,^38^,^39^,^40^,^41^, where solute atoms diffuse and stick to dislocations, thereby pinning them in place. The more strongly a dislocation is pinned in place, the larger the stress that is required to mobilize the dislocation again. In our theory, the increased pinning and subsequent weakening if a dislocation breaks free from the pinning atoms is modeled by the strengthening of the weakspots and their weakening behavior upon slipping.

However, there is a key difference between our proposed strengthening mechanism for medium-entropy alloys (MEAs) and high-entropy alloys (HEAs) and the conventional DSA mechanism in the PLC effect. In MEAs and HEAs, the “pinning” of dislocations originates from abundant sources of solutes in the whole-solute matrix. *As a result, the strengthening* (*healing*) *rate, Λ, in the PLC effect for MEAs and HEAs is much higher than that in the conventional PLC effect*.

#### Model Predictions for the serration statistics

The model predicts the serration statistics for experiments in which the material is sheared at a slow strain-rate, *Ω*, either by tensile deformation or by compression. The model can be solved in the mean-field theory (MFT) approximation^42^, where the elastic interactions between the weak spots are approximated to be constant in space. Tools from the theory of phase transitions, such as the renormalization group, and simulations can be used to show that MFT gives the correct scaling behavior of the avalanche statistics on long length scales and time scales^42^,^44^,^45^. As detailed below, MFT predicts that for the finite weakening, ε, *i.e*., in a particular temperature and strain-rate regime, the material produces small and large avalanches. The small avalanches are much smaller in extent than the sample size, so that they are not affected by sample boundaries. In contrast, the large avalanches span the system, touching opposite sample boundaries. The large avalanches recur almost periodically, while the small avalanches occur at random times between the large avalanches. The large avalanches are predicted to show crack-like scaling^42^, *S* ~ *R*^*3*^, of the avalanche size, *S*, with its radius, *R*. The reason for the crack-like scaling behavior is that for the large slips, the model predicts that the total amount of slip at a point grows with the radius *R* of the slipping area, so that the total slip integrated over the entire area scales as *R*^*3*^. The large avalanches typically have a Gaussian size-distribution. Their average avalanche size grows with increasing weakening, ε. In contrast, for small avalanches, the model predicts *S* ~ *R*^*2*^. The reason is that small avalanches are more pulse-like, where the amount of slip in each slipping patch is fixed, *i.e*., it does not grow with the radius, R, of the slipping area^42^. In this case, the distribution, *D*(*S*), of the small slip-avalanche sizes, *S*, scales as


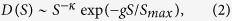


where *κ* = *1.5*, *g* is a material-specific constant, and *S*_*max*_ is the expected cutoff size of the power-law scaling regime of *D*(*S*)^42^. The maximum avalanche size, *S*_*max*_ ~ *1/ε*^*2*^, decreases with the inverse square of the weakening parameter, *ε*. The complementary cumulative distribution function (CCDF), defined as 
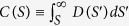
, gives the probability that the size of a slip avalanche is larger than *S*. For the smaller slips that occur between the almost periodically-recurring largest slips, Equation (2) implies that *C*(*S*) scales as^42^,^45^


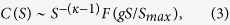


where again, *κ* = *1.5*, and


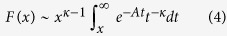


is another quickly-decaying scaling function^42^,^45^, with *A* as a material-dependent parameter.

Below we summarize the MFT predictions for the slip statistics in the following three cases: (1) the deformation rate is slow, compared to the healing rate of the weak spots (in this case, the dislocations are strongly pinned), (2) the deformation rate is comparable to the healing rate [in this case the pinning of the dislocations by solute atoms is not as strong as in case (1)], and (3) the deformation rate is fast, compared to the healing rate (in this case, the solute atoms hardly have time to pin the dislocations). As explained below, these three cases correspond to different values of the weakening parameter, *ε*, in the model:

1. *For sufficiently-slow imposed strain-rates* (*such that the deformation rate is slow, compared to the healing rate*), the weak spots have sufficient time to fully re-heal after a slip avalanche. Consequently, the weakening, ε, upon slipping again is *large* in this case, and the small avalanches may be almost undetectably small, because S_max_ ~ 1/ε^2^ is small. Strong weakening also implies almost regularly-recurring large slips with a narrowly-peaked size distribution. In this strain-rate regime, only the periodically-recurring large slips are large enough to be detectable^42^. The corresponding stress-strain curves resemble “type-C PLC-bands”^25^,^30^,^38^,^39^,^40^,^41^, characterized by the almost periodically-recurring large slips (see Fig. 1).
2. *In the intermediate strain-rate regime* (*such that the deformation rate is comparable to the healing rate*), there is less time to pin the dislocations between avalanches. Consequently, the dislocations cannot be pinned as strongly before they move again, making the weakening, *ε*, smaller than in the first case. As a result, the largest *small* avalanche, *S*_*max*_ ~ *1/ε*^*2*^, is larger than in the previous case. In contrast, the almost periodically-recurring *large* avalanches are smaller than before. Additionally, these *large* avalanches occur less regularly than in case (1). Because the small avalanches are now larger, they are more easily detectable in this regime than in the first case^42^. The resulting stress-strain curves resemble type-B PLC-bands^25^,^30^,^38^,^39^,^40^,^41^, which is usually characterized by smaller system-spanning (*large*) slips that occur less regularly than in the above type-C PLC-bands, and along with many broadly-distributed *small* slips (see Fig. 1).
3. *In the fast strain-rate regime* (*such that the deformation rate is fast, compared to the healing rate*), there is very little time for the dislocations to be re-pinned after they slip. Consequently, the model assumes that in this regime, the effective weakening, *ε*, *is small.* The reason is that a slipping weak spot frees a dislocation from a much weaker pinning potential than in the other cases, because the weak spots have effectively no time to re-strengthen between slips. (Remember that the weakening does not reflect the overall strength of the material but how much the system weakens, relative to its overall strength just before an avalanche.) In this case, one may expect *S*_*max*_ ~ *1/ε*^*2*^ to be large. As we show below, in the experiments presented here, this regime is observed at the lowest tested temperatures, where the pinning atoms move more slowly than in the other two regimes, and, therefore, do not have sufficient time to strongly pin the dislocations. The resulting reduced pinning of the dislocations can reduce *S*_*max*_ to a smaller value^46^,^47^ than in the other regimes, because the dislocations typically slip less in a slip event if they have previously been pinned less strongly, i.e., if they start to slip at effectively lower stresses. The distribution of slip sizes is given by Equations (2) and (3) above and no periodic large events are observed^42^. For sufficiently large *S*_*max*_ the distribution will show power law scaling in the range *S* < *S*_*max*_ (see Equation (2)). Near and above slip-size *S*_*max*_, the size distribution is predicted to have a noticeable exponentially decaying roll-off given by the scaling function in Equation (2) and (3). This case is most closely related to the slip statistics in single-element crystals, where there is no weakening effect, i.e., *ε* = *0*. The resulting stress-strain curves resemble those of type-A PLC-bands^25^,^30^,^38^,^39^,^40^ (see Fig. 1) with irregularly-occurring smaller slips that are broadly distributed in size.

#### Tuning between the three regimes

In summary, the weakening, *ε*, is the key parameter that determines whether the serrations in the model are of type-A, B, or C PLC-bands. The weakening, ε, is determined by how much time the atoms have to pin the dislocations, before they slip again in the next avalanche. Consequently (in analogy to the time-temperature superposition in polymers^43^), the weakening, ε, is predicted to grow with the ratio, *Λ/Ω*, of the healing rate, *Λ*, and the strain-rate, *Ω*. In experiments, this ratio can be tuned by changing the strain-rate, the temperature, or the number of elements in the material.

The healing rate, *Λ*, is affected by both the temperature and the number of elements in the material. It can be tuned as follows: At lower temperatures, atoms move more slowly to re-pin the dislocations, leading to a lower healing rate, *Λ*. For Arrhenius-activated healing processes (i.e., at the lower temperatures), *Λ* ~ exp(*−B*/*T*), where *B* is a material-dependent constant. (Note that at very high temperatures, *Λ* is expected to strongly decrease again, because the thermal fluctuations are so strong that they prevent any dislocation pinning.) Larger numbers of elements are also expected to lead to faster healing rates, as they provide more mobile atoms that can move to stick to the dislocations to pin them more strongly in place.

In summary, by tuning the temperature, strain-rate, and/or the number of elements in the alloy, the serration behavior can be tuned between the three different regimes discussed above.

For increasing temperature, the healing rate, *Λ*, first increases and, then, decreases again. The weakening, ε ~ Λ/Ω, follows the same trend. As a result, the model predicts that at fixed, low strain-rates, *Ω*, smaller serrations with a *broad power-law s*ize distribution are observed only in a specific temperature regime. This temperature range is set by the condition that that ε must be sufficiently small so that the power-law-distributed events of the maximum size, S_max_ ~ 1/ε^2^, are large enough to be detected in the experiments (such as in types-A and B PLC-bands). As a consequence, *the model predicts that for increasing temperature, the serrated stress-strain curves are expected to move from type-A to B to C PLC-bands.* At temperatures far above the serration regime, all serrations are washed out by thermal fluctuations, and the stress-strain curves look smooth. At temperatures far below this regime, serrations are not observed either, because of the lack of sufficient pinning of the dislocations by the surrounding atoms on experimental time scales in this regime. The range of temperatures that shows a broad range of serration sizes is predicted to be material specific, since the healing rate is determined by the material-specific diffusion constants of atoms.

*The model also predicts the dependence on the strain-rate and composition of the temperature window in which serrations are observed*: The ratio, *Λ/Ω*, of the healing rate to the strain-rate is the key parameter determining the temperature range where serrations are observed. We already saw that *Λ* decreases with decreasing temperatures. The model, then, predicts that for smaller strain-rates, serrations are observed for a smaller window of healing rates, and, thus, for a smaller range of temperatures. The dependence of the serration statistics on *Λ/Ω* implies that at fixed temperatures and increased strain-rates, HEAs are predicted to move from type-C to B to A PLC-bands, to no serrations at all.

Increasing the number of components also increases the healing rate and, thus, the ratio (*Λ/Ω*), thereby widening the temperature range over which serrations are observed. In the following section, we compare the theoretical predictions to the experimental results.

#### Comparison of experiments to model predictions

We find that the observed stress-strain curves for the type-A PLC-bands agree with the model predictions of random sequences of slips with a wide range of sizes. Similarly, the stress-strain curves for types-B and C bands are also consistent with the model predictions in that they display almost-periodically recurring large slips, as discussed in the Model Section (see Fig. 1). Figure 1 shows that for increasing temperatures, the stress-strain curves of the same HEA move from type-A, to B, to C PLC-bands, as predicted by the model.

The model also predicts that for increasing strain-rate, the PLC-bands move from type C to B to A to none (see the Model Section above). Table 2 lists the results for the CoCrFeMnNi alloy tested with various strain-rates. It can be seen that as the strain-rate increases, the trend of serration types is consistent with the model prediction. For example, the combination of *Ω* = 10^−5^/s and 275 °C gives a PLC effect with type B, while that of *Ω* = 10^−4^/s and 275 °C gives no PLC effect. And at 600 °C one finds PLC-bands that move from type-C to B to A as the strain-rate is increased, as predicted (see Table 2).

The slip-size distributions are also consistent with the model predictions. Figure 2 shows that for materials with the type-A or B deformation, in a certain temperature range the CCDF of the stress-drop sizes, *S*, follows a power-law, *S*^−*κ*^, over a wide range of sizes, *S*, consistent with the model predictions of Equations (2) and (3). The theoretical value, *κ* = *1.5*, predicted by the mean-field model for type-A bands is consistent with the observations (see Fig. 2c). For other slip size distributions of type-A or B bands the temperature range is picked such that mostly the roll-off function is observed, which is also consistent with Equations (2) and (3) (see Fig. 2a,b).

Table 1 shows the effect of composition on the slip statistics: in the tested temperature range, Ni and CoNi (LEAs) have no serration phenomena, but CoFeNi (MEA), CoCrFeNi (MEA), and CoCrFeMnNi (HEA) display serration behaviors in certain temperature ranges. CoCrFeMnNi shows serration characteristics in a temperature range similar to that of CoFeNi but broader than that of CoFeNi. This trend suggests that the structure of CoCrFeMnNi allows atoms to pin the dislocations at lower temperatures and prevents larger thermal vibrations from destroying the pinning effect. In addition, the quinary alloy shows the most pronounced PLC effect (see Fig. 1), even though it has the slowest diffusion rate of atoms because of the sluggish diffusion effect^48^. The above effects of the number of elements on the serration behavior are consistent with the expectations obtained form the model predictions, i.e. increasing the number of elements increases the range of temperatures over which serrations are observed.

Furthermore, we find that the PLC effect of CoCrFeMnNi remains at greater strain-rates up to 10^−2^/s for higher temperatures, except for the experiments at 300 °C. *This fact demonstrates that the pinning of dislocations is fast. Thus, the pinning mechanism of such alloys differs from that in conventional alloys with low solute contents, for which the pinning of dislocation is much slower.* The difference in the pinning mechanisms will be quantified in future work.

The striking agreement of the serration statistics and the observed PLC-bands with the predictions of the model for slip avalanches shows that the serrations can be explained as avalanches of slipping weak spots. In MEAs and HEAs, the weak spots (i.e., the pinned dislocations) weaken upon slipping and re-strengthen (or *in-situ* pin) at a relatively-fast healing rate between avalanches. In addition, as temperature is increased, the healing rate, *Λ*, of the weak spots increases to a maximum at a certain temperature, and, then, quickly decreases. As a result, six phenomena are observed that are consistent with the proposed model:

1. Higher temperatures create a larger probability of overcoming the energy barrier to successfully rearrange the atoms and lock the dislocations (*i.e.*, the healing rate, *Λ*, of the weak spots increases with temperature). However, a limiting temperature is reached, when the thermal vibration of atoms is too large to settle down the effective locking (*i.e.*, this limiting temperature defines the temperature where the healing rate, *Λ*, reaches a maximum). The experiments show that the working temperature range to observe serration behavior at a strain-rate of 10^−4^/s covers from around 300 °C to 620 °C for the present quinary alloy (CoCrFeMnNi). At temperatures above 650 °C, no serrations are observed prior to the critical failure (see Table 1).
2. Increasing the number of sources and the size differences of various atomic pairs (the atomic radii in FCC structures are: R_Ni_ = 1.25 Å, R_Co_ = 1.25 Å, R_Fe_ = 1.27 Å, R_Cr_ = 1.28 Å, and R_Mn_ = 1.26 Å)^49^ in the lattice causes a lower and wider temperature range in which serration behavior is observed (and in which the healing rate, *Λ*, increases and decreases again with temperature). This trend explains why the quinary alloy has the largest range, while the ternary alloy has the narrowest range. Ni and CoNi alloys are lacking sufficient sources and size differences and, accordingly, show no PLC effect (see Table 1).
3. Lowering the strain-rate can decrease the high-temperature limit of the serration behavior because lower strain-rates allow atoms to have more time to successfully lock the dislocations. Table 2 demonstrates this effect. A strain-rate of 1 × 10^−5^/s produces serration behavior at 300 °C, whereas a strain-rate of 1 × 10^−4^/s does not.
4. The PLC effect of CoCrFeMnNi remains at higher strain-rates up to 10^−2^/s for higher temperatures except for 300 °C. This fact demonstrates that the pinning of dislocations is fast (which implies the healing rates, *Λ*, are fast), compared to conventional PLC-pinning mechanisms.
5. As shown in Fig. 2c for materials with type-A or B PLC-deformation bands, within a certain temperature range, the size distribution of the sizes, *S*, of the *small* stress drops follows a power law, *S*^−*κ*^, with an exponent, *κ*, that is consistent with the theoretical value of *κ* = 1.5. The range over which a power law can be observed depends on temperature, strain-rate, and material composition. The model predicts that the power law distribution is multiplied by an exponentially-quickly decaying roll-off function, which cuts off the power law at a largest slip size, *S*_*max*_ for the largest slip sizes^42^. Figure 2 shows that the slip size, *S*_*max*_, where the exponential roll-off is observed depends on the temperature and the number of elements in the material. For type-B bands in a certain temperature range, *S*_*max*_, is so small that the power law range is not visible and only the exponential roll-off is seen because it dominates over the power law decay for the measured slip sizes (Fig. 2b). The temperature dependence of the roll-off is consistent with the model, because in the model, the roll-off depends on the weakening, *ε*, in the material, which in turn depends on temperature, strain-rate and material composition, as explained in the model section. The slip statistics for the type-C band behavior are also consistent with the model, as explained in the model section. In summary, Fig. 2 shows that both types-A and B bands typically have the predicted broad distribution of *small* events whose size distribution typically follows a power law multiplied by a temperature-, and composition dependent exponential roll-off for the larger sizes, while type-C bands have a sharply-peaked distribution of *large* slips indicated by the steep drop-off at the high end of the CCDF in Fig. 2. As predicted, type-B bands may also have some, though fewer, of those narrowly-distributed *large* slips (as indicated by a similar sharp drop at the high end of the CCDF in Fig. 2c), while type-A bands generally have none in Fig. 2c. Also, the largest slips for the type-B bands are smaller than the largest slips for the type-C bands (see Fig. 2c). All of these features are consistent with the predicted temperature-, strain-rate-, and composition-dependent weakening ε in the model. The model predicts that the size of the largest slips increases with the amount of weakening, and, as discussed in the Model Section, the weakening is larger for type-C bands than for type-B bands. Therefore, the model predicts that the largest slips are larger for type-C bands than type-B bands, as is observed in the experiments (see Fig. 2).
6. As argued in the Model Section, for a given HEA material, *similar slip statistics (such as type-A bands) are expected for temperatures, T, and strain-rates, Ω, chosen such that Λ/Ω ~* exp*(−B/T)/ Ω = constant, or equivalently*

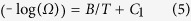


Here *B* and *C*_*1*_ are material-specific constants. In Fig. 3, we plot (−log(*Ω*)) versus *1/T* for the temperatures, *T*, in Table 2 that are just below the transition from type-A to B bands. For each of these temperatures and strain-rates, similar slip statistics are observed. (The data points extracted from Table 2 are (*Ω, T*) = (*10*^−*4*^*/s, 300* °*C*), (*Ω, T*) = (*10*^−*3*^*/s, 400* °*C*)*, and* (*Ω, T*) = (*10*^−*2*^*/s, 600* °*C*). As predicted, within the accuracy of the temperature step size in the experiments, the data points in Fig. 3 fall on a straight line, consistent with three predictions: (1) the validity of Equation (5), (2) the underlying prediction of the time-temperature superposition^43^, and (3) the prediction of Arrhenius-type activation processes describing the healing rates in this temperature and strain-rate regime.

## Discussion

The effect of changing temperatures and adding elements to alloys ranging from FCC-LEAs to FCC-HEAs has been studied to investigate the PLC effect at a strain-rate of 10^−4^/s. The effect of varying the strain-rates from 1 × 10^−5^ to 1 × 10^−2^/s on the serration behavior was examined. The PLC effect is observed in a specific range of temperatures and strain-rates for MEAs and HEAs, and not in LEAs representing the conventional substitutional alloys.

As shown in the above six observations, the experimental results agree with the model introduced above. Although the pinning mechanism of such alloys is different from that in conventional alloys with low solute contents, their serrations can be explained as avalanches of slipping weak spots. The main difference is that in MEAs and HEAs, the weak spots (or dislocations) weaken upon slipping and re-strengthen (or *in-situ* pin) at a relatively fast healing rate between avalanches, compared to conventional alloys. In addition, the PLC effect is more pronounced in HEAs than in MEAs.

The present study provides a basic understanding of the deformation mechanisms by modeling, predicting, and measuring the observed statistics of slips in LEAs, MEAs, and HEAs, and their temperature, strain-rate, and composition dependence.

These new HEA materials have important high-temperature applications for technologies, such as next-generation power plants. The present research significantly advances our fundamental understanding of the mechanical behavior of these advanced alloys^1^,^2^,^3^,^4^,^5^,^6^,^7^,^8^,^9^,^10^,^11^,^12^,^13^,^14^,^15^,^16^,^17^,^18^. We show new experiments and statistical theory on the intermittent deformation of slowly-deformed LEAs, MEAs, and HEAs with a single FCC structure. In a specific temperature and strain-rate regime, intermittent slips are observed. The slip statistics are related to the more general phenomena of crackling noise and/or avalanches in many other systems, including earthquakes, nanocrystals, conventional alloys, bulk metallic glasses, magnetic materials, granular materials, and decision making processes^3^,^6^,^42^,^44^,^45^,^46^,^47^. The present integrated experimental and theoretical approach may, thus, be applied to the challenges of general materials evaluation and alloy design with prescribed slip-statistics properties in a desired temperature range for next-generation technologies, while fostering a deeper scientific understanding of the new materials class of HEAs.

The methods of this paper are broadly applicable to many disciplines, including materials science, physics, mechanical science and engineering, earth science, and aerospace engineering.

## Conclusions

In the present series of experiments on LEAs (Ni and CoNi), MEAs (CoFeNi and CoCrFeNi), and HEA (CoCrFeMnNi) with a single FCC structure, serration phenomena are only found in MEAs and HEA. The experimental results are in remarkable agreement with the predictions of a simple MFT model. The model predicts that the critical exponent, *κ*, and more generally, the scaling behavior of the slip-size distributions, are universal, i.e., independent of material details. The model predicts the observed serration statistics as a function of temperature, strain-rate, and composition. It thereby provides the first quantitative explanation for the observed serration statistics and their dependence on temperature, strain-rate, and composition of LEAs, MEAs, and HEAs. The observed serration statistics confirm that weak spots, which slip along slip planes (when a sufficiently-large shear stress is applied) trigger other weak spots to slip, resulting in slip avalanches typically detected as sudden stress drops. During slip avalanches, the participating weak spots are weakened from their original strength to a lower strength, but they are re-strengthened during the time intervals between slip avalanches. Re-strengthening or healing of weak spots between slip avalanches is temperature-, strain-rate-, and composition-dependent. More abundant sources of solute atoms give higher healing rates. The healing rate reaches a maximum at a material-specific temperature and strain-rate. For the temperature regime in which type-A PLC-bands are observed, the healing rate has an Arrhenius-type temperature dependence. For a given HEA material, similar slip statistics are observed for experiments at similar ratios of the healing rate to strain-rate, thereby revealing an underlying deformation mechanism that is analogous to the time-temperature superposition in polymers^43^.

The results provide us with a new understanding of the deformation mechanisms of LEAs, MEAs, and HEAs and a simple mechanism for the PLC effect in general. The observed serration statistics are related to the more general phenomenon of crackling noise and/or avalanches in many other systems, ranging from HEAs, conventional alloys, bulk metallic glasses, nanomaterials and magnets to earthquakes and other systems^3^,^6^,^42^,^44^,^45^,^46^,^47^. The results of this study can be used for (1) materials evaluation and (2) alloys design with prescribed serration properties in desired temperature and strain-rate ranges.

## Methods

**Figure 4:**
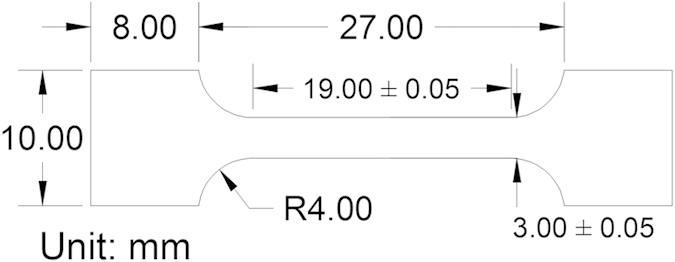
Dimensions of the tensile specimen used in all experiments. All numbers are in mm. R indicates the radius of curvature.

The five experimental alloys were vacuum arc-melted from the starting elements with purities higher than 99.9 weight percent (wt. %). Each sample was re-melted and re-solidified at least four times to assure the uniform composition under the protection of argon. The alloys were, then, cast into a slab of dimensions, 10 mm × 20 mm × 40 mm. After surface machining on cast slabs, a cold-rolling reduction of 65% was performed from the initial thickness of 8 mm to a thickness of 3.2 mm. The alloys were subsequently subjected to a homogenization treatment at 1,100 °C for 6 hours and follow-on quenching in water. The sheets were wire cut by the electrical discharge machine into tensile specimens with dimensions shown in Fig. 4. The thickness of the specimen is 1 mm, and any oxidation debris on the top and bottom surface layers has been removed. The five alloys were tension-tested by an Instron 4505 testing machine at a strain-rate of 10^−4^/s and a data-acquisition frequency of 20 Hz. Each alloy was tested at different temperatures:, 300 °C, 400 °C, 500 °C, 600 °C, and 700 °C. The CoCrFeMnNi alloy had additional samples tested at 275 °C, 325 °C, 350 °C, 375 °C, 620 °C, and 650 °C, with additional strain-rates of 10^−2^/s, 10^−3^/s, and 10^−5^/s for detailed investigations. For each condition, 3 or 4 test specimens were used. The stress versus time data was recorded during the experiment. The complementary cumulative distribution function, *C*(*S*), of stress-drop sizes for each condition was extracted from a representative curve of three or four measured curves^45^,^46^.

## Additional Information

**How to cite this article**: Carroll, R. *et al.* Experiments and Model for Serration Statistics in Low-Entropy, Medium-Entropy, and High-Entropy Alloys. *Sci. Rep.* **5**, 16997; doi: 10.1038/srep16997 (2015).

## Supplementary Material

Supplementary Information

## Figures and Tables

**Table 1:** List of each alloy tested along with each temperature.

Alloy	Temperature (°C)	Serration Behavior	PLC-Band Type
Ni	300	None	None
	400	None	None
	500	None	None
	600	None	None
	700	None	None
CoNi	300	None	None
	400	None	None
	500	None	None
	600	None	None
	700	None	None
CoFeNi	300	None	None
	400	Yes	B
	500	Yes	B
	600	None	None
	700	None	None
CoCrFeNi	300	Yes	B
	400	Yes	B
	500	Yes	B
	600	Yes	C
	700	None	None
CoCrFeMnNi	275	None	None
	300	Yes	A/B
	325	Yes	A
	350	Yes	B
	375	Yes	B
	400	Yes	B
	500	Yes	B
	600	Yes	C
	620	Yes	C
	650	None	None
	700	None	None

The presence of serration behavior and types-A, B, and C PLC-band are indicated. “None” represents no serration or no PLC-band. The tension experiments were conducted at a strain-rate of 1 × 10^−4^/s.

**Table 2:** List of the CoCrFeMnNi alloy tested with various strain-rates.

Strain-rate	Temperature (°C)	Serration Behavior	PLC-Band Type
1 × 10^−2^/s	300	None	None
	400	Yes	A
	500	Yes	A
	600	Yes	A
1 × 10^−3^/s	300	Yes	A
	400	Yes	A
	500	Yes	B
	600	Yes	B
1 × 10^−4^/s	300	Yes	A
	400	Yes	B
	500	Yes	B
	600	Yes	C

The presence of serration behavior and types-A, B, and C PLC-bands are indicated. “None” represents no serration or no PLC-band.
