# Supplementary material for: Experiments and Model for Serration Statistics in Low-Entropy, Medium-Entropy, and High-Entropy Alloys
Source: Sci Rep. 2015 Nov 23;5:16997. doi: 10.1038/srep16997 (PMC4655315; doi:10.1038/srep16997)
Supplement: Supplementary Information [file srep16997-s1.doc]

Supplementary Information:

Experiments and Model for Serration Statistics in Low-Entropy, Medium-Entropy, and High-Entropy Alloys

Robert Carroll^1^, Chi Lee^2^, Che-Wei Tsai^2^, Jien-Wei Yeh^2,*^, James Antonaglia^1^, Braden A. W. Brinkman^1,‡^, Michael LeBlanc^1^, Xie Xie^3^, Shuying Chen^3^, Peter K. Liaw^3,*^, and Karin A. Dahmen^1,*^

1. University of Illinois at Urbana-Champaign, Department of Physics, 1110 West Green Street, Urbana, IL 61801

2. National Tsing Hua University, Department of Materials Science and Engineering, Hsinchu, 30013, Taiwan

3. The University of Tennessee-Knoxville, Department of Materials Science and Engineering, Knoxville, TN 37996.

* Corresponding authors.

^‡^ Now at the University of Washington, Department of Applied Mathematics, Seattle, WA 98195.

Otto et al. [S1] investigated tensile properties and micro-structural evolution during deformation of a CoCrFeMnNi equi-atomic alloy with a solo face-centered-cubic (FCC) structure.

Tensile testing at 400°C yielded serrations in the tensile stress-strain curves of both fine-grained and coarse-grained specimens (grain sizes: 4.4 and 155 µm, respectively). This alloy also displayed large strain hardening and great tensile elongation of at least 50% at room temperature, and almost double strength with higher ductility at - 196°C. At small strains (< 2.4%), the deformation is evidenced by the planar slip of partial dislocations, indicating low stacking fault energy. At a strain higher than 20%, the dislocation cell structure forms for 20°C, whereas nano-twins develop at - 196°C. However, the serration phenomena were not examined in detail.

**Stress-Strain Curves trend lines**

**
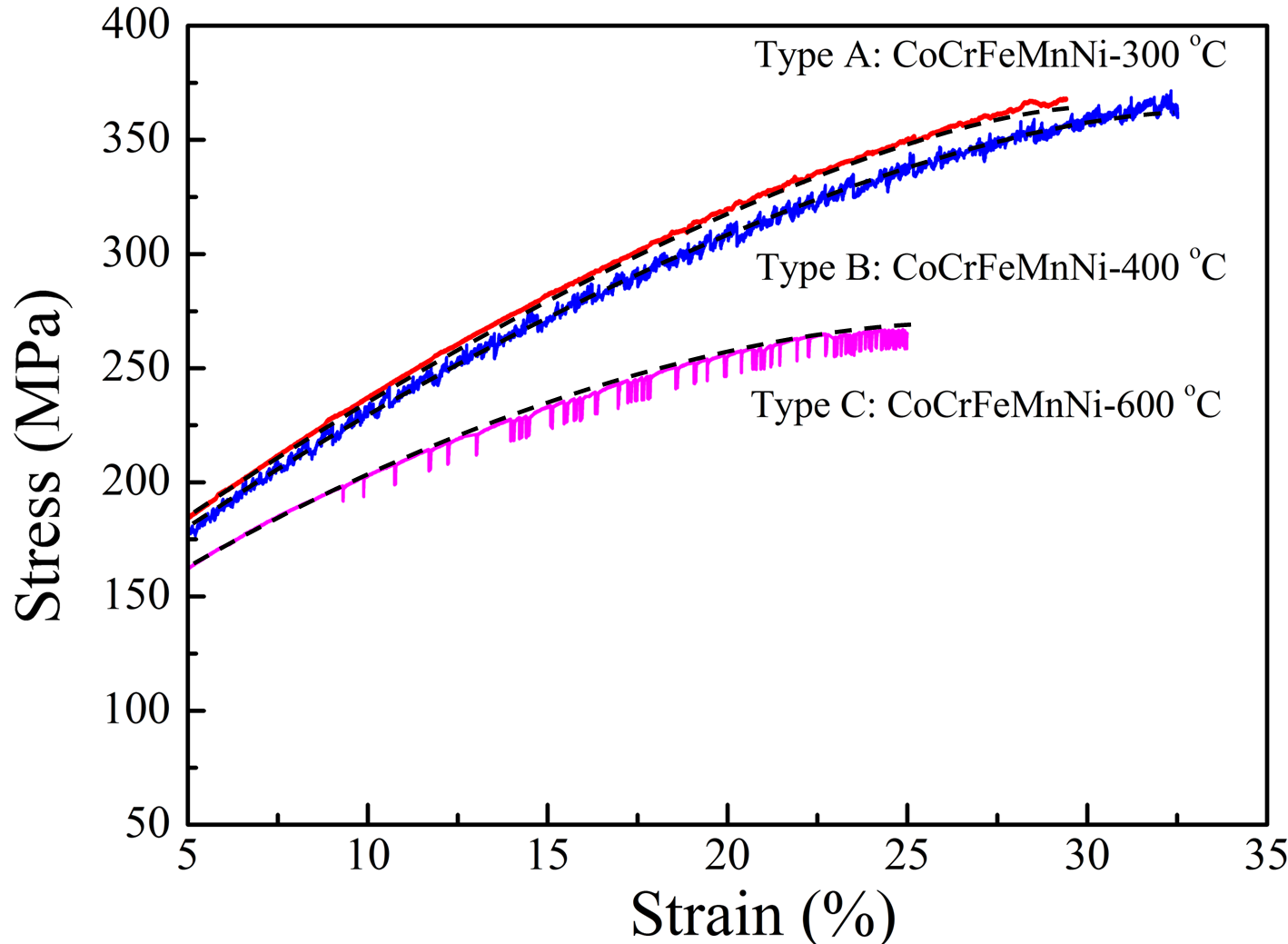
**

**Figure S1:** *Close-ups of experimentally-measured stress-strain curves with average trend lines (same data as shown in Figure 1 in the main paper). (a) Type-A example from CoCrFeMnNi, 300°C, at a strain-rate of 10^-4^/s. (b) Type-B example from CoCrFeNi, 400°C, at a strain-rate of 10^-4^/s. (c) Type-C example from CoCrFeNi, 600°C, at a strain-rate of 10^-4^/s. The type-A curve shows several abrupt rises, each followed by a small stress drop (serration) very close to the dashed curve, which is plotted to show the trend of the general level of the stress-strain curves. It generally occurs in the low-temperature (high strain-rate) part of the dynamic-strain-aging (DSA) regime. Type-B curves quickly oscillate about the general level (i.e., the dashed curve) of the stress-strain curve. They occur at higher temperatures and lower strain-rates of the DSA regime and also develop from type-A with increasing strain. Type-C serrations are stress drops that occur below the general level (very close to the dashed curve showing the trend of the general level) of the flow curve. They occur at higher temperatures and lower strain-rates than types A and B.*

**More details on Portevin LeChatellier bands of types-A, B, and C:**

In the literature on the serration behavior of conventional materials and from the viewpoint of dynamic strain aging (DSA), Types-A, B, and C serrations in the tensile stress-strain curves can be defined as follows [S2-S11], as shown in Figure S1:

Type-A serrations are stress fluctuations with small amplitudes from repeated deformation bands, initiating at one end of the specimen and propagating to the other end along the loading direction. These fluctuations are considered as locking serrations, characterized by an abrupt rise followed by a drop above the general level (the trend line for the red curve in Figure S1) of the flow stress. They occur in the low temperature (high strain-rate) part of the DSA regime.

Type-B serrations, corresponding to the hopping propagation of localized bands in the loading direction, are oscillations around the general level (the trend line for the blue curve in Figure S1) of the stress-strain curve that occur in quick succession due to discontinuous band propagation arising from the DSA of the moving dislocations within the band. (This trend contrasts with the continuous propagation of the band that occurs after initiation with a Type-A load drop). Type-B band appears at various places in the specimen, propagates for short distances, and then disappears.

Type-C serrations are yield drops that occur below the general level (the trend line for the pink curve in Figure S1) of the flow curve and are, therefore, considered to be due to dislocation unlocking. When band propagation becomes impossible, deformation proceeds through the formation of localized non-propagating bands appearing at random locations along the specimen axis, resulting in Type-C serrations wherein each stress decrement indicates the formation of a non-propagating deformation band. They occur at higher temperatures and lower strain-rates than in the case of Types-A and B serrations.

In the literature on conventional alloys, such as Ni-based alloys in the temperature range of 500K to 798K, serration types change from A, to A + B, B, and C [S12]; Hastelloy X in the temperature range from 673K to 923K with serration types changing from A, to A + B, B, and C [S13]; 316L(N) austenitic stainless steel in the temperature range from 723K to 923K with serration types changing from A, to A+B and A+C [S14].

In the present study, the serration behavior was studied in a new class of materials called high-entropy alloys [S1,S15-S31] in light of the mean-field theory [S32].

**References**

S1. Otto, F. *et al.* The influences of temperature and microstructure on the tensile properties of a CoCrFeMnNi high-entropy alloy. *Acta Mater.* **61**, 5743-5755 (2013).

S2. Kubin, L., Fressengeas, C. & Ananthakrishna, G. Collective behaviour of dislocations in plasticity. *Dislocations in solids* **11**, 101-192 (2002).

S3. Ananthakrishna, G., Noronha, S. J., Fressengeas, C. & Kubin, L. P. Crossover from chaotic to self-organized critical dynamics in jerky flow of single crystals. *Physical review. E, Statistical, nonlinear, and soft matter physics* **60**, 5455-5462 (1999).

S4. Bharathi, M. S., Lebyodkin, M., Ananthakrishna, G., Fressengeas, C. & Kubin, L. P. The hidden order behind jerky flow. *Acta Mater.* **50**, 2813-2824 (2002).

S5. Hähner, P. & Rizzi, E. On the kinematics of Portevin–Le Chatelier bands: theoretical and numerical modelling. *Acta Mater.* **51**, 3385-3397 (2003).

S6. Kok, S. *et al.* Spatial coupling in jerky flow using polycrystal plasticity. *Acta Mater.* **51**, 3651-3662 (2003).

S7. Rizzi, E. & Hahner, P. On the Portevin-Le Chatelier effect: theoretical modeling and numerical results. *International Journal of Plasticity* **20**, 121-165 (2004).

S8. Zhang, Q., Jiang, Z., Jiang, H., Chen, Z. & Wu, X. On the propagation and pulsation of Portevin-Le Chatelier deformation bands: An experimental study with digital speckle pattern metrology. *International journal of plasticity* **21**, 2150-2173 (2005).

S9. Jiang, H. *et al.* Three types of Portevin–Le Chatelier effects: experiment and modelling. *Acta Mater.* **55**, 2219-2228 (2007).

S10. Ait-Amokhtar, H. & Fressengeas, C. Crossover from continuous to discontinuous propagation in the Portevin–Le Chatelier effect. *Acta Mater.* **58**, 1342-1349 (2010).

S11. Rodriguez, P. Serrated plastic flow. *Bull. Mater. Sci.* **6**, 653-663 (1984).

S12. Hale, C. L., Rollings, W. S. & Weaver, M. L. Activation energy calculations for discontinuous yielding in Inconel 718SPF. *Materials Science and Engineering A* **300**, 153-164 (2001).

S13. Sakthivel, T. *et al.* Effect of temperature and strain rate on serrated flow behaviour of Hastelloy X. *Materials Science and Engineering A* **534**, 580-587 (2012).

S14. Choudhary, B. Activation energy for serrated flow in type 316L (N) austenitic stainless steel. *Materials Science and Engineering: A* **603**, 160-168 (2014).

S15. Gludovatz, B. *et al.* A fracture-resistant high-entropy alloy for cryogenic applications. *Science* **345**, 1153-1158 (2014).

S16. Cantor, B., Chang, I. T. H., Knight, P. & Vincent, A. J. B. Microstructural development in equiatomic multicomponent alloys. *Materials Science and Engineering A* **375**, 213-218 (2004).

S17. Zhang, Y. *et al.* Microstructures and properties of high-entropy alloys. *Prog. Mater. Sci.* **61**, 1-93 (2014).

S18. Santodonato, L. J. *et al.* Deviation from high-entropy configurations in the atomic distributions of a multi-principal-element alloy. *Nat. Commun.* **6**, 5964 (2015).

S19. Senkov, O. N., Miller, J. D., Miracle, D. B. & Woodward, C. Accelerated exploration of multi-principal element alloys with solid solution phases. *Nat. Commun.* **6**, 6529 (2015).

S20. Antonaglia, J. *et al.* Temperature effects on deformation and serration behavior of high-entropy alloys (HEAs). *JOM* **66**, 2002-2008 (2014).

S21. Zhang, Y., Zhou, Y. J., Lin, J. P., Chen, G. L. & Liaw, P. K. Solid-solution phase formation rules for multi-component alloys. *Advanced Engineering Materials* **10**, 534-538 (2008).

S22. Senkov, O. N., Wilks, G. B., Miracle, D. B., Chuang, C. P. & Liaw, P. K. Refractory high-entropy alloys. *Intermetallics* **18**, 1758-1765 (2010).

S23. Guo, S., Ng, C., Lu, J. & Liu, C. T. Effect of valence electron concentration on stability of fcc or bcc phase in high entropy alloys. *Journal of Applied Physics* **109**, 103505 (2011).

S24. Hemphill, M. A. *et al.* Fatigue behavior of Al0.5CoCrCuFeNi high entropy alloys. *Acta Mater.* **60**, 5723-5734 (2012).

S25. Zhang, Y., Zuo, T. T., Cheng, Y. Q. & Liaw, P. K. High-entropy alloys with high saturation magnetization, electrical resistivity, and malleability. *Scientific Reports* **3**, 1455 (2013).

S26. Yeh, J. W. (Ed.). High Entropy Alloys [Special Issue]. *Entropy* (2013).

S27. Yeh, J. W. *et al.* Nanostructured high-entropy alloys with multiple principal elements: Novel alloy design concepts and outcomes. *Advanced Engineering Materials* **6**, 299-303 (2004).

S28. Wu, J. M., Lin, S. J., Yeh, J. W., Chen, S. K. & Huang, Y. S. Adhesive wear behavior of AlxCoCrCuFeNi high-entropy alloys as a function of aluminum content. *Wear* **261**, 513-519 (2006).

S29. Yeh, J. W., Chen, Y. L., Lin, S. J. & Chen, S. K. in *Advanced Structural Materials III* Vol. 560 *Materials Science Forum* (eds H. B. Ramirez *et al.*), 1-9 (2007).

S30. Yeh, J. W. Recent progress in high-entropy alloys. *Annales De Chimie-Science Des Materiaux* **31**, 633-648 (2006).

S31. Yeh, J. W. Alloy design strategies and future trends in high-entropy alloys. *JOM* **65**, 1759-1771 (2013).

S32. Dahmen, K. A., Ben-Zion, Y. & Uhl, J. T. Micromechanical model for deformation in solids with universal predictions for stress-strain curves and slip avalanches. *Physical Review Letters* **102**, 175501 (2009).
